# Retrospective Study on the Occurrence of Antibodies against *Coxiella burnetii* in Dogs from Central Italy

**DOI:** 10.3390/pathogens9121068

**Published:** 2020-12-20

**Authors:** Valentina Virginia Ebani

**Affiliations:** 1 Department of Veterinary Science, University of Pisa, Viale delle Piagge 2, 56124 Pisa, Italy; valentina.virginia.ebani@unipi.it; 2 Centre for Climate Change Impact, University of Pisa, Via del Borghetto 80, 56124 Pisa, Italy

**Keywords:** *Coxiella burnetii*, dogs, IFAT, serology, zoonosis

## Abstract

*Coxiella burnetii,* a cause of infection in humans and several animal species, is transmitted through inhalations and oral route but also tick bites. Its spreading in ruminants has been largely investigated, whereas data about the occurrence of this infection in canine population are scanty. In this retrospective study, blood serum samples of 516 dogs were tested by indirect immunofluorescence assay to detect antibodies against *C. burnetii*; 42 (8.13%) were positive with titers ranging from 1:64 to 1:512. The highest seroprevalences were detected in dogs aged > 5 years, employed in hunting activity and living in a peri-urban/rural environment. Diagnosis for *C. burnetii* infection should be always carried out in bitches with reproductive disorders. Moreover, in view of the zoonotic impact of this infection, asymptomatic dogs exposed to ticks’ bites and/or to contact with infected farm animals should be checked, too.

## 1. Introduction

*Coxiella burnetii* is a Gram-negative, intracellular obligate bacterium responsible for the infectious disease called Q Fever, which affects domestic and wild animals and humans.

The agent has a widespread geographical distribution; currently, it has not been registered only in New Zealand and Antarctica [1]. It is largely investigated in domestic ruminants worldwide. In fact, cattle, sheep, and goats are known as the main reservoirs of *C. burnetii* and the main source of infection for humans [2]. In recent years, an increasing number of other animal species have been reported to shed the bacterium, including domestic mammals, marine mammals, reptiles, and birds [3]. Among domestic animals, also dogs and cats have been suggested to be involved in the Q Fever epidemiology [4,5,6].

Q Fever is classified as a tick-borne infection because the agent can be transmitted by ticks mainly of the genera *Ixodes, Rhipicephalus, Dermacentor,* and *Amblyomma* [7]. However, the epidemiology is traditionally related to transmission through the ingestion of contaminated animal food, mainly milk and dairy products, and the inhalation of aerosol particles contaminated with the pathogen [4].

*C. burnetii* is known as a cause of reproductive disorders in farm ruminants that excrete the bacteria with placenta, aborted fetus, lochiations, and milk. Furthermore, infected animals can excrete coxiellae in urine and feces, too [4].

*C. burnetii*-infected dogs and cats can develop asymptomatic forms, and pregnant females may have abortions as well. DNA of the agent was found in different canine specimens, suggesting that dogs may be a source of infections for their owners and other animals [8,9].

Even though the role of dogs in Q Fever epidemiology raises the interest of researchers in view of the One Health perspective, data about the spreading of this infection in canine populations are scant. In particular, investigations carried out in Italy were mainly performed on farm animals and wildlife [10,11,12,13,14,15,16], but studies on companion animals are very limited [17,18,19,20].

For this reason, the present study had the purpose of verifying the occurrence of antibodies against *C. burnetii* in domestic dogs living in different environments and conditions in Central Italy during the period from 2015 to 2019.

## 2. Results

Among the 516 tested sera, 42 were positive with a mean prevalence of 8.13%. A statistically higher value of positive reactions was observed in dogs employed in hunting activity (14.45%). High percentages of positive dogs were found in animals aged more than 5 years (10.4%) and living in peri-urban/rural environments (11.17%) (Table 1).

The positive sera had antibody titers ranging from 1:64 to 1:512; in detail, 27 (64.28%) samples were positive at 1:64 titer, 11 (26.19%) were positive at 1:128, 3 (7.14%) were positive at 1:256 and 1 was positive (2.38%) at 1:512. All positive sera reacted against the phase II antigen.

Data concerning gender, age, environment, attitude, and antibody titer for each dog resulted positive to *C. burnetii* are reported in Table 2.

## 3. Discussion

The incidence of the arthropod-borne infections has been increasing in several parts of the world as a result of the global climate change. The spreading of these infections, of veterinary and human concern, has become an important threat in Italy, too, involving the health of companion and farm animals, as well as people.

Q Fever is an important zoonosis classified as an arthropod-borne disease because it is also transmitted through tick bites. It is largely investigated in domestic ruminants worldwide, whereas no numerous data about its spreading in companion animals are available.

The 8.13% mean seroprevalence detected in the present survey showed that the tested dogs of different age and from different environment developed antibodies against *C. burnetii*.

A statistically relevant difference was observed comparing the percentages detected among hunting (14.45%) and companion dogs (6.92%).

Dogs employed in hunting activity could contract *C. burnetii* through the ingestion of infected wild preys, considering that this pathogen infects mammals in wild environments, such as rodents, hares, and wild rabbit [21,22]. However, the tested dogs that frequented the forest environment could be exposed to tick bites and consequently to arthropod-borne pathogens. These results are corroborated by those obtained in a previous molecular study that found *C. burnetii* DNA in blood of dogs involved in hunting activity in Central Italy with a 5% prevalence [19].

Ticks could have transmitted *C. burnetii* to companion dogs, too. The lower seroprevalence detected in this category could be related to the fact that dogs kept as pets usually are less exposed to ticks, because they are regularly checked by owners and live in domestic environment where ticks are present only occasionally.

It was not possible to know if the dogs resulted seropositive had contact with infected animals, but it probably can be excluded that they had contact with *C. burnetii*-infected farm animals and their products.

All positive dogs of this study had antibodies against the phase II antigen of *C. burnetii,* suggesting that they had no acute infection at the blood sampling time. In fact, reactivity to phase I and II antigens indicates acute and past infection, respectively. No information about why dogs were submitted to blood collection as well as about clinical signs referable to *C. burnetii* infection at the sampling time were available; thus, the results of the present serological investigation could be influenced by the undefined tested population.

An indirect immunofluorescence antibody test (IFAT) and enzyme-linked immunosorbent assay (ELISA) are frequently used in veterinary and human medicine for the diagnosis of Q Fever. In fact, although the complement fixation test, reported by OIE (Office International des Epizooties) as a reference test, is highly specific, IFAT and ELISA have higher sensitivity and are able to detect antibodies earlier [6].

Some positive results could be due to cross-reactions with other pathogens. Possible cross-reactions between *C. burnetii* and *Bartonella henselae*, *Bartonella quintana* [23], *Legionella micdadei* [24], and *Legionella pneumophila* [25] have been suspected in humans. However, information about cross-reactivity between *C. burnetii* and other pathogens in dogs is not available.

It is difficult to compare the results of this investigation with those obtained in other studies, because there are deep differences in relation to several factors, such as whether the tests employed detect positive animals, canine populations, environment in which dogs lived, and Q Fever-epidemiological geographical situation.

Previous serological studies carried out in Italian dogs found different prevalences ranging from 0.87% to 8% [17,18], whereas a survey to detect *C. burnetii* in canine placenta and aborted fetuses did not find positive samples [20].

A recent study in Montenegro found antibodies to *C. burnetii* in three dogs (1.2%), two from the public dog shelter and one from the coastal area. This low seroprevalence has been explained by the fact that Q fever was not endemic or enzootic in the investigated area [9].

Similarly, a low (2.9%) seroprevalence was detected in dogs living in South Korea [26] and Q fever seroprevalence in feral dogs in Iraq was 5.5% in the period from 2007 to 2008 [27].

Conversely, a 26.6% seroprevalence was detected in canine population in Australia, but the authors, who did not find *C. burnetii* DNA in whole blood, reproductive tissue or vaginal/preputial swabs, hypothesized that dogs are not important as source of infections for humans [28].

Mtshali and coworkers analyzed ticks, identified as *R. sanguineus, Haemaphysalis elliptica,* and *Amblyomma hebraeum* and collected from dogs in South Africa; *C. burnetii* DNA was found in 41% of the tested ticks [29]. A 10% molecular prevalence was found in domestic dogs in Zambia [30].

A past study carried out in a hospital population of companion animals and livestock in California during 1973–1975 found a high overall seroprevalence (53%) for *C. burnetii* in the canine population. More in detail, 346 (48%) of 724 hospitalized dogs and 208 (66%) of 316 stray dogs had antibodies against *C. burnetii.* These results could be related to a widespread presence of the pathogen, as also suggested by the relevant percentages of seroprevalence detected in cats (9%), horses (26%), and cattle (32%) [31]. Furthermore, an earlier survey found a 63% seroprevalence among canids, coyotes, and foxes, in wildlife areas of California [31].

Although the risk of human *C. burnetii* infection related to dogs is directly proportional to the spreading of this pathogen among canine population, persons—mainly owners, veterinarians, and breeders—can become infected even through direct or indirect contact with only one *C. burnetii* infected dog.

An outbreak of Q Fever has been reported in a family where *C. burnetii* pneumonia developed in all three members exposed to an infected parturient bitch that gave birth to four pups; three died shortly after birth, and the fourth died within 24 h of birth [32].

Disease in humans can be sometimes asymptomatic, but it often determines acute undifferentiated febrile illness with the possibility of focal manifestations, such as hepatitis and pneumonia. Acute forms can progress to chronic forms, with chronic fatigue syndrome and endocarditis, mainly in individuals predisposed by valvular heart disease or immunodeficiency [33].

## 4. Material and Methods

### 4.1. Animals

The study was performed testing canine blood serum samples collected during previous years for other purpose and kept in collection at −20 °C. The sera were sampled from privately owned dogs from different areas of Central Italy during the period January 2015–December 2019.

Collected sera were selected if all the following data were available: data of sampling, animal age (<1 year old, 1–5 years old, >5 years old) and gender, environment in which dogs lived (urban, peri-urban/rural), and animal attitude (hunting or companion dogs). Moreover, the sera of dogs that were under antibiotic treatment at the sampling time were excluded. No data about possible clinical signs referable to Q fever were available. A total of 516 canine sera were selected for this retrospective study. Data concerning gender, age, environment and attitude for each tested dog are reported in Table S1.

#### Ethical Statement

No dogs were submitted to the blood collection exclusively for this study. In fact, the present investigation was carried out using sera previously collected for other purposes by collaborating veterinarians during clinical visits.

### 4.2. Serological Analyses

The indirect immunofluorescence antibody test (IFAT) was executed using commercial IFAT slides specific for *Coxiella burnetii* antigens (Fuller Laboratories Fullerton, CA, USA). As reported by the producer, the slides were coated with separate phase I and phase II antigens in each well.

Blood sera were diluted 1:64 in phosphate-buffered saline (PBS, pH 7.2), which was considered as the cut-off dilution, and tested following the procedures reported by the IFAT slides producer. Positive samples were successively two-fold serially diluted to determine the endpoint antibody titer. Scores from 1 to 4 were assigned to the intensity of specific fluorescence and the antibody titer was defined as the major dilution with a ≥2 score.

### 4.3. Statistical Analysis

Statistical evaluation was carried out by the χ^2^ test to analyze the results of serological tests in relation to gender, age, attitude of the tested dogs, and environment in which they lived. Values of *p* < 0.05 were considered significant.

## 5. Conclusions

The detection of antibodies against *C. burnetii* in the examined canine populations showed that the animals were exposed to this pathogen. Infected bitches may develop abortion, whereas male dogs are often asymptomatic. In all cases, infected dogs excreting coxiellae in fetuses, urine, and feces [8] may be source of infection for humans.

The diagnosis of Q fever is routinely performed in farm ruminants with reproductive disorders, whereas it is rarely requested for dogs. Differential diagnosis in case of abortion in bitches should always include microbiological diagnosis for Q Fever. Furthermore, considering that *C. burnetii*-infected dogs can be asymptomatic, periodical control for this infection should be carried out. In particular, dogs living in peri-urban and rural environments, as well as those employed in hunting activity, should be submitted to serological and/or molecular diagnosis for arthopod-borne infections including Q Fever.

In view of the importance of a correct diagnosis and considering that serological tests, on which diagnosis primarily relies, cannot be translated directly from one species to another [6], further studies are necessary to standardize the diagnostic tests to use in dogs.

## Figures and Tables

**Table 1:** Serological results in relation to age, gender, attitude, and environment of the tested dogs.

Category		Study Population	Positive Dogs (%)	*p* Value
gender	male	302	25 (8.27)	0.89
	female	214	17 (7.94)	
age	<1	21	0	0.05
	1–5	197	11 (5.58)	
	>5	298	31 (10.40)	
attitude	companion	433	30 (6.92)	0.02
	hunting	83	12 (14.45)	
environment	urban	346	23 (6.64)	0.07
	peri-urban/rural	170	19 (11.17)	
Total		516	42 (8.13)	

**Table 2:** Data concerning gender, age, environment, attitude, and antibody titer for each dog resulted positive to *Coxiella burnetii* by an indirect immunofluorescence test.

Dog (ID)	Gender	Age (Years)	Attitude	Environment	Antibody Titer
1 (27)	male	1–5	companion	urban	1:128
2 (38)	male	>5	companion	urban	1:64
3 (65)	male	1–5	companion	urban	1:64
4 (71)	female	>5	companion	urban	1:64
5 (78)	male	>5	companion	urban	1:128
6 (101)	female	1–5	companion	peri-urban/rural	1:256
7 (129)	female	1–5	companion	peri-urban/rural	1:64
8 (145)	male	>5	companion	urban	1:64
9 (166)	male	>5	companion	urban	1:64
10 (179)	female	>5	companion	peri-urban/rural	1:128
11 (201)	female	>5	companion	peri-urban/rural	1:64
12 (223)	male	1–5	companion	peri-urban/rural	1:128
13 (238)	male	1–5	companion	urban	1:128
14 (277)	male	>5	companion	urban	1:64
15 (311)	female	>5	companion	urban	1:64
16 (357)	male	>5	companion	urban	1:64
17 (388)	female	>5	companion	peri-urban/rural	1:128
18 (391)	female	>5	companion	urban	1:128
19 (431)	female	>5	companion	peri-urban/rural	1:64
20 (446)	male	1–5	companion	urban	1:64
21 (448)	male	1–5	companion	urban	1:64
22 (455)	male	>5	companion	peri-urban/rural	1:256
23 (466)	male	>5	companion	peri-urban/rural	1:64
24 (475)	female	1–5	companion	urban	1:64
25 (486)	male	>5	companion	urban	1:64
26 (497)	female	>5	companion	peri-urban/rural	1:64
27 (498)	female	>5	companion	peri-urban/rural	1:64
28 (500)	male	>5	companion	urban	1:128
29 (502)	male	>5	companion	urban	1:64
30 (505)	female	>5	companion	urban	1:64
31 (205)	male	1–5	hunting	urban	1:128
32 (206)	male	>5	hunting	urban	1:64
33 (234)	female	>5	hunting	peri-urban/rural	1:256
34 (235)	female	>5	hunting	peri-urban/rural	1:64
35 (236)	male	>5	hunting	peri-urban/rural	1:128
36 (378)	female	1–5	hunting	peri-urban/rural	1:64
37 (379)	male	>5	hunting	peri-urban/rural	1:64
38 (380)	male	>5	hunting	peri-urban/rural	1:512
39 (421)	female	>5	hunting	peri-urban/rural	1:64
40 (424)	male	>5	hunting	peri-urban/rural	1:64
41 (511)	male	>5	hunting	urban	1:64
42 (513)	male	>5	hunting	urban	1:128

Legend—ID: identification number.

## Supplementary Materials

The following are available online at https://www.mdpi.com/2076-0817/9/12/1068/s1, Table S1: Data concerning gender, age, environment, attitude for each tested dog.
